# Development of a Novel Motor Imagery Control Technique and Application in a Gaming Environment

**DOI:** 10.1155/2017/5863512

**Published:** 2017-05-09

**Authors:** Ting Li, Jinhua Zhang, Tao Xue, Baozeng Wang

**Affiliations:** ^1^School of Computer Science, Xi'an Polytechnic University, Xi'an, China; ^2^State Key Laboratory for Manufacturing Systems Engineering, School of Mechanical Engineering, Xi'an Jiaotong University, Xi'an, China

## Abstract

We present a methodology for a hybrid brain-computer interface (BCI) system, with the recognition of motor imagery (MI) based on EEG and blink EOG signals. We tested the BCI system in a 3D Tetris and an analogous 2D game playing environment. To enhance player's BCI control ability, the study focused on feature extraction from EEG and control strategy supporting Game-BCI system operation. We compared the numerical differences between spatial features extracted with common spatial pattern (CSP) and the proposed multifeature extraction. To demonstrate the effectiveness of 3D game environment at enhancing player's event-related desynchronization (ERD) and event-related synchronization (ERS) production ability, we set the 2D Screen Game as the comparison experiment. According to a series of statistical results, the group performing MI in the 3D Tetris environment showed more significant improvements in generating MI-associated ERD/ERS. Analysis results of game-score indicated that the players' scores presented an obvious uptrend in 3D Tetris environment but did not show an obvious downward trend in 2D Screen Game. It suggested that the immersive and rich-control environment for MI would improve the associated mental imagery and enhance MI-based BCI skills.

## 1. Introduction

Gamification is the application of game-design elements and game principles in nongame contexts [1, 2] in attempts to improve user engagement [3, 4], organizational productivity [5], physical exercise [6], and traffic violations [7], among others [8]. With the development of gamification, video game has been playing important roles in a variety of environments, from marketing [9–11] to inspiration [12] to health [13] and education [14–16]. Moreover, many areas of neuroscience (supported by Open Fund of Key Laboratory of Electronic Equipment Structure Design (Ministry of Education) in Xidian University (EESD-OF-201401)) have used video games as tools to study the effectiveness of electroencephalography in measuring visual fatigue, Internet game addiction, and remedies for motion sickness [17–22], which makes video game studies span a wide range of areas and clinical applications. Video game environment involves human interaction with a user interface to generate visual feedback on a video device or commands to control a robot. The feedback expresses user's motion, emotional and some other intentions. Those feedbacks to the nervous system close the control loop of the man-machine system [23]. In some applications, control of devices by means of neural processes (e.g., via EEG signals) can replace or enhance motor control. A particularly important application is rehabilitation, particularly with individuals who may have mobility restrictions.

Brain-computer interface (BCI) is a direct communication pathway between an enhanced or wired brain and an external device [17]. As a particular class of human-machine interface, BCI has spurred a surge of research interest. This technology serves the demands of activities from rehabilitation to assistive technology to daily civil field. Noninvasive EEG-based technologies and interfaces have been used for a much broader variety of applications. As the most discussed BCI control method, motor imagery (MI) offers an essential basis for the development of BCIs [18]. Researchers have set up demonstrations on the feasibility of motor imagery brain-computer interface (MI-BCI) for different applications, especially in rehabilitation. From the perspectives of temporal regularities, neural encoding rules, and biomechanical constraints, researchers have uncovered many significant properties of motor imagery [18]. However, current MI-based BCIs are limited in imagination of only four movements: left hand, right hand, feet, and tongue [19]. It is still challenging to design an effective and flexible BCI system for complex controls in practical applications since the number of control commands in the BCI system is strictly limited [20]. Although EEG-based interfaces are easy to wear and do not require surgery, they have relatively poor spatial resolution and cannot effectively use higher-frequency signals. Most MI-BCI systems rely on temporal, spectral, and spatial features to distinguish different MI patterns. Another substantial barrier to using EEG as a BCI was the extensive training required before users can work the technology [21, 22, 24].

One of important factors improving the efficiency of MI-based BCI is the experiment paradigm, because the motivational experiment paradigms for MI provide more enlightenment and guidance for users to study neural control of movement. Allison et al. [25] proposed that if BCI methods are effective, gamers will be the most active testers. Van Erp and colleagues [26] predicted that, beyond rehabilitation uses, video game and entertainment would be the most promising application of BCIs. In the near future, games seem likely to be a very potent direction for application of BCI technology [27]. Video display has been a primary and important experimental tool in the BCI field, such as imaging hands or other parts of body moving according to certain static cue shown on the computer screen. “Static cue” is the original instruction pattern appearing in MI research. The thinking about this pattern is to simplify environmental stimuli, so that the participants can concentrate on mental tasks. This kind of experiment paradigm suits users without too much experience to gain MI skills, but lack of interestingness and inspiration for extensive training. People live in complicated and dense environments. They pay attention to objects which are important or interesting to them. Intuitively, it would seem that combining MI and BCI should provide more flexible environments, leading to enhancement of users' sense of stimuli. Current 3D video games provide abundant and rich information (stimulus and feedback) to immerse players in the game scenarios. The interaction patterns of these games include powerful move-enabled control and accurate feedback of players' operations. So we deduce that MI-BCI with game environment can connect the player to the action in the game in a more realistic and involving way.

How can a BCI experimental paradigm be more attractive? Though games can provide strong motivation for practicing and achieving better control for users within a rehabilitation system, the amount of information interaction during gaming should be adjusted to a proper range. The idealized experimental environments would not only be attractive to players (to reduce distraction) but also enhance the performing efficiency of motor imagery and help inexperienced users. So experimental objectives should be the core design principles of experimental design; meanwhile, content and forms should be vivid and rich. Marshall et al. designed a system to encourage rapid generation of mental commands and enhance the user's experience in motor imagery-based BCI [28]. Lalor et al. [29] refitted a game paradigm by introducing traditional steady-state visual evoked potential (SSVEP) BCI to improve user's concentration. That form of BCI used the SSVEP generated in response to phase-reversing checkerboard patterns to achieve binary control in a visually elaborate immersive 3D Mind Balance game [30]. The software converted brain signals relevant to two classes of motor imagery (left and right hand movement) to pinball game commands for control of left and right paddles [31]. In addition, studies have demonstrated examples of BCI applications developed in other game environments, such as Pacman [32], Tetris [33], and World of Warcraft [34]. The systems mentioned above mainly provided binary control, and players had a low level of operation, which would weaken the entertainment and immersion of BCI system. To resolve this problem, we must enable Game-BCI systems to provide more training functions. In order to make video game program in which BCI control is feasible, researchers need to simplify the original program to achieve the application with game-design elements and game principles in nongame contexts [1, 2].

Based on the reasons mentioned above, we conjectured that an immersive 3D game environment could promote characteristic brain state generation in the context of motor imagery. We implemented in a Game-BCI system for 3D Tetris game playing, which was a hybrid brain-computer interface (BCI) system, with the recognition of motor imagery based on EEG and blink EOG signals. A hybrid BCI system usually contained two or more types of BCI systems. And BCI system also could be combined with another system which is not BCI-based, for example, combining a BCI system with an electromyogram- (EMG-) based system. The research on hybrid BCI has been a mainstream research direction in BCI field. Many works [35–38] with great academic value stated the important ideas for the development of hybrid BCI.

The main content of paper can be divided into five parts. In Sections 2.4.2 and 3.2, the method of multifeature extraction for extracting features of MI EEG was developed and tested separately. The mechanism in translation from classification results of MI to the control commands in 3D Tetris game was explained in Section 2.5. Then in the work reported in this paper, to help demonstrate the effectiveness of the system, and as a point of comparison with the 3D environment, we also applied the new system in a 2D game scenario. Through all this work we expected to prove the effectiveness of gamification strategy for enhancing players' BCI control abilities.

## 2. Materials and Methods

### 2.1. Participants

Ten players (3 females and 7 males) without previous BCI experience participated in the experiment voluntarily. All these players were right-handed, and their mean age was 24.6 years with a standard deviation of 3.3 years. All these players were conducted in accordance with the highest ethical standards of Xi'an Jiaotong University and signed the declaration file to declare they volunteered for the research experiment.

### 2.2. Apparatus

**Figure 1:**
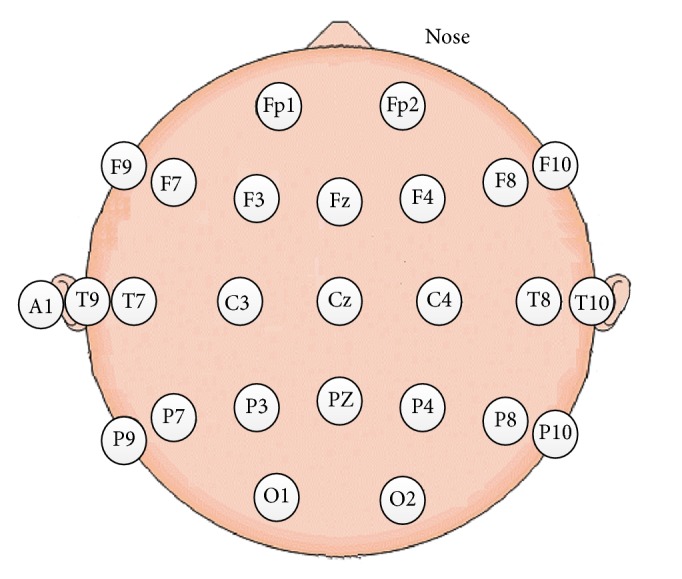
Positions of 25-channel EEG electrodes on players' scalps.

We used the 40-channel NuAmps system (America, Neuroscan Co.) to acquire EEG and EOG data. The system collected and transformed data using the TCP/IP (Transmission Control Protocol/Internet Protocol) protocol. The sampling rate was 1000 Hz. EEG data was recorded from 25 scalp electrodes, placed as shown in Figure 1. The reference electrode was on the left ear (Electrode A1). For all electrodes, the impedance was <5 kΩ. Four additional electrodes were used to record horizontal and vertical EOG. Scan 4.5 performed online EOG artifact rejection. A 50 Hz notch filter suppressed line noise.

### 2.3. Procedures

#### 2.3.1. Motor Imagery Training

**Figure 2:**
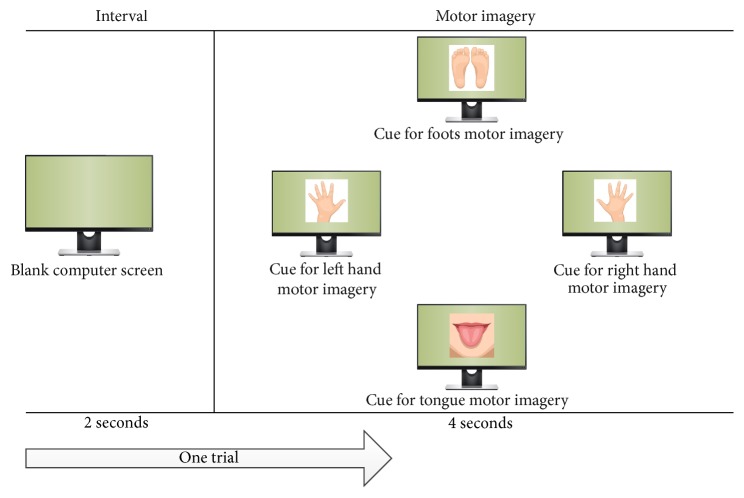
The flow of one single trial for MI training.

Before 3D Tetris game playing, all players went through a process of MI training. We familiarized them with the feeling of performing of four kinds of motor imagery. In the MI training phase, the participant sat in a comfortable armchair in front of a computer screen (Dell S2316 M LED monitor, maximum resolution: 1920 × 1080) for sixty centimeters. We instructed participants to imagine right hand, left hand, foot, and tongue movements corresponding to visual cues showed on the computer screen. Each trial began with a 2 sec interval in which the screen was blank. Then players took 4 secs to do motor imagery. The screen then was again blanked to begin the next trial. The flow of one single trial for MI training was showed in Figure 2. We collected data for each participant in two sessions over two days. Each session contained two runs, in each of which the four types of cue were displayed 15 times in a randomized order, giving a total of 240 trials for each participant. Each session lasted approximately sixteen minutes.

#### 2.3.2. 3D Tetris Game Playing

**Figure 3:**
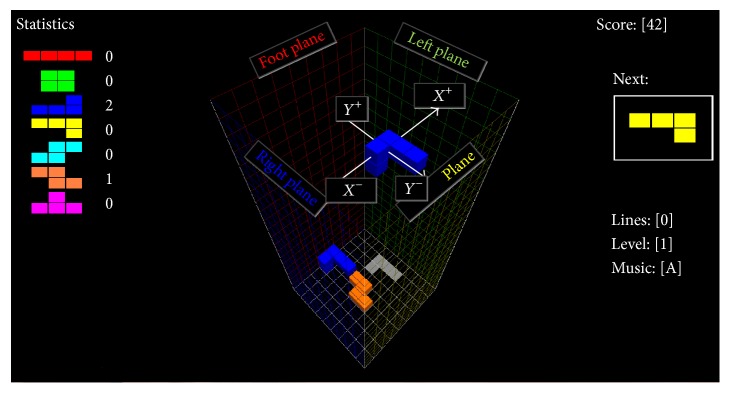
3D Tetris Scene.

In the 3D Tetris experiment, we divided the 10 players into two equal groups: One group experienced the traditional asynchronous BCI paradigm and the other group experienced the 3D Tetris paradigm. The 3D Tetris procedure was a puzzle game that used a three-dimensional playing field, as opposed to the traditional two dimensional pattern mentioned in literature [39]. In the 3D Tetris displays, three-dimensional block groups constructed of small single cubic blocks arranged in different shapes keep falling into a 3D space from the top of the screen. The player adjusted the position and moving direction of these block groups such that they fell into a pattern forming a larger complete shape with no gaps. The 3D space was a cuboid with an open top and closed bottom (see Figure 3). The bottom plane appeared as a white grid. The four standing planes displayed as a red grid, green grid, yellow grid, and blue grid. Here, we used names associated with the semantic meanings of MI cues appearing in MI training phase to label the four standing planes, namely, Foot Plane, Left Plane, Tongue Plane, and Right Plane (see Figure 3).

During game playing, we used the names of standing planes to label the direction of motion of the block groups. In coordinates of block group, Foot Plane represents *y*-axis positive direction. Left Plane represents *x*-axis positive direction. Tongue Plane represents *y*-axis negative direction. Right Plane represents *x*-axis negative direction. “Moving to Foot Plane” meant that if the Game-BCI system produced an identification result of the player's mental state as “MI of foot motion,” then the block group would move one unit length in the direction of the Foot Plane. The unit length of a block group move was determined by the original 3D Tetris program and was not changed in this research. This 3D space contained 20 vertical layers. When players filled one layer with falling block groups, that layer disappeared, and the player earned one score unit. If blocks stacked over a given layer, but gaps remained in the layer, the number of layers went down by one. The game was over when the final layer was lost. In our experimental paradigm, players used four kinds of MI commands to control the movement direction of block groups and used two kinds of blink EOG commands to rotate the block groups. With the control commands translated from EOG, the falling three-dimensional block groups could be rotated about any of the three coordinate axes. As a block fell, its shadow appeared at the bottom of the 3D space; the shadow indicated where the block would land, if it continued to fall without the player's intervention. The BCI control details are explained in Section 2.5.

### 2.4. Data Handling Procedures

**Figure 4:**
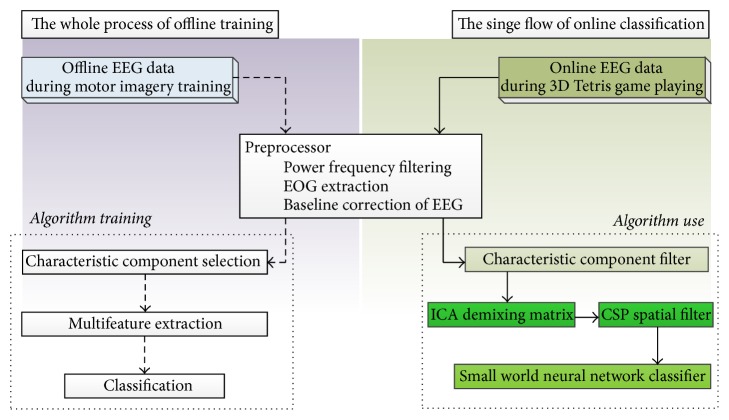
The illustration of data handling procedures.

In this research, the data processing showed in Figure 4 contained two sections: offline data analysis and algorithm training and online control. The processing of online control would use the characteristic component filter, ICA demixing matrix, CSP spatial filter, and Small World Neural Network Classifier, which were obtained from the processing of offline data analysis and algorithm training.

In both offline calculation and online control, preprocessing steps included power frequency filtering, EOG extraction, and baseline correction of EEG. We used all EEG data collected in the MI training phrase in feature component extraction and algorithm training (classification and feature extraction). Trial data striping and feature component extraction only occurred in offline calculation.

#### 2.4.1. Characteristic Component

Ten players participated in the MI training phrase. For each player, we collected 240 trials of EEG data, giving 60 trials for each kind of motor imagery. For each kind of motor imagery, we averagely separated the data of each player into 6 parts. Each part contained 10 trials EEG data related to given kind of motor imagery. For each trial of EEG data, we applied CAR spatial filtering to each of the 25 data channels firstly and then selected the data recorded after 4 seconds of the MI cue presentation. Chebyshev I Bandpass filters of order 10 were used for extracting multiband data, with the range from 0 Hz to 60 Hz and frequency band 2 Hz wide. Subsequently, the filtered data was separated into components labeled by frequency band and electrode.

We calculated the spectral power for each selected component and the average *R*-squared values of components, which were labeled by the same frequency band and electrode, but by different MI categories. *R*-squared values provide a measure for the amount to which a particular EEG feature is influenced by the subject's task (e.g., hand versus foot imagery) [40]. It is an evaluation index used to determine which brain signal feature differ the most between two particular tasks. Then it is necessary to verify whether the feature in question is consistent with the sensorimotor rhythm's known properties to avoid misconfiguration due to EEG artifacts, other noises, or random effects [40]. According to the *R*-squared values among the four kinds of motor imagery, we noted frequencies and electrodes of the components with the top 10 largest *R*-squared values. Depending on the *R*-squared values, the most significant components were found. Then according to the properties of ERD and ERS patterns appearing in the process of MI [41], we screened all selected components and picked up the most suitable ones for the classification of motor imagery. All selected components were used to train the algorithms for feature extraction and classification.

#### 2.4.2. Multifeature Extraction

In this investigation, we proposed a method of multifeature extraction. That procedure combined independent component analysis and common spatial patterns in a renovated mode.

*(1) Independent Component Analysis Keeping Temporal Structure of EEG Signals*. The first step was to conduct an independent component analysis (ICA), keeping the temporal structure of the EEG signal. EEG is a kind of mixed signal, generated by underlying components of brain activity in many different regions and recorded from a number of locations on the scalp. To find the original components of brain activity and define the brain states, our task was to reveal the underlying brain activity by separating the mixed signal into components associated with their independent sources. The traditional ICA algorithm identifies temporally independent sources in multichannel EEG data. However, on account of the strong noise and the ignorance of the temporal structure of EEG signals, the algorithm fails to remove EEG noise from EEG waveforms. Therefore, we formulated a new method for independent sources extraction, which could pass on the time pattern from the original signals to the statistically independent components. This computational method adopted multivariable autoregression to represent the original temporal structures. All regression coefficients were estimated by least square methods. Concerning the measure of the independence, we analyzed the residuals in the autoregression model, instead of estimating source signals, by minimizing the mutual information between them, and modified the unmixing matrix by the natural gradient algorithm.

In this method, we described the time pattern of the sources by a stationary autoregression model

(1) $$\begin{array}{c} S_{t}=\overset{P}{\underset{K=1}{\sum }}A_{K}S_{t-K}+\Phi_{t} \end{array}$$

in which *S*_*t*_ = [*S*_*t*_^1^, *S*_*t*_^2^,…,*S*_*t*_^*M*^]^*T*^ is a vector including *M* source signals, *A*_*K*_ stands for the regression coefficients, and Φ_*t*_ = [*e*_1_(*t*), *e*_2_(*t*),…,*e*_*M*_(*t*)]^*T*^ is the residual vector. Considering the course of regression coefficients estimation, (1) could be rewritten as

(2) $$\begin{array}{c} V_{t}=AU_{t}+E_{t},\;t=1,\ldots ,N, \end{array}$$

where *A* = (*A*_1_, *A*_2_,…, *A*_*p*_) ∈ *R*^*M*×*M*×*P*^ is the coefficient matrix. And *U*_*t*_ = (*S*_*t*−1_,…,*S*_*t*−*p*_)^*T*^ ∈ *R*^*M*×*P*^, *V*_*t*_ = *S*_*t*_. Then (2) approximates a multilinear regression model. That meant that we could take *P* values in the source signals before *t* time point as a time-sampling to be an independent variable of the linear system and the value at *t* time point as a predicted value to the dependent variable accordingly.

The assumption which was important to the least squares estimation method used in linear regression analysis required residuals to have the statistic characteristics *e* ~ *N*_*M*_(*O*_*M*×1_, *σ*^2^_*M*×*M*_). When *e*_*i*_ kept statistical independence from others, the linear system had normal random distribution. So there was no serial correlation between all independent variables, expressed as

(3) Eei=0,covei,ej=σ2,i=j0,i≠j,i,j=1,2,…,M.

Based on this equivalence relationship, the correlation among all independent components in the temporal model was measured with minimization of mutual information.

*(2) One-versus-Rest CSP*. The next step is common spatial pattern (CSP) extraction. The procedure discussed above explains our approach to temporal feature extraction. We aimed to find an algorithm for spatial feature discovery, which could use ICA components as inputs. The main trick in the binary case is that the CSP algorithm yields a simultaneous diagonalization of both covariance matrices whose eigenvalues sum to one. We adopted a CSP method termed one-versus-rest (OVR), which enabled the CSP in the ordinary sense to handle a multiclassification problem. In this algorithm, each model corresponding to one kind of MI would produce a spatial filter versus other models. The details of the CSP algorithm are in Appendix.

In order to compare the multifeature extraction to traditional CSP, we define two computation processes. First, we let the feature components be the processing objects of the CSP spatial filter directly. The spatial features obtained in this way are called cspW_Data. Second, we let the feature components go through the independent component analysis and then used CSP spatial filtering to process those independent components. The spatial features obtained with the method of multifeature extraction were called cspW_IC. By comparing the quantitative differences between spatial feature cspW_Data and cspW_IC, we tried to demonstrate the effectiveness of the method of multifeature extraction.

#### 2.4.3. Classification

In this work, we used the small world neural network (SWNN), discussed in previous research [42], as the classifier. The SWNN was constructed based on a multilayered feedforward perception model, with the weight adjustment mechanism involving both backpropagation and cutting and rewiring connections. The SWNN included one input layer, one output layer, and 10 hidden layers with eight neurons in each hidden layer. The dimension of a given CSP feature determined the number of neurons in the input layer. The output layer contained four neurons. We assigned the hard-limit transfer function [43] to the output layer, which made the SWNN output a 4-bit gray code (right hand motor imagery: 0001, light hand motor imagery: 0010, foots motor imagery: 0100, and tongue motor imagery: 1000).

During classifier training, we defined four 4-bit gray codes to stand for the four kinds of motor imagery. If the SWNN produced a 4-bit gray code different from the four desired ones, we defined this brain state as idle. There was no “idle” data collected in the MI training phase, but players would exhibit idle states during game playing. The features extracted from idle state data would not produce a 4-bit gray code to be one of the four predefined ones.

### 2.5. Control Strategy

In the original 3D Tetris game, the coordinate system of the 3D space and the local coordinate system of the block group were predefined. So the BCI system just took advantage of the original definition of the coordinate systems to adjust the movement and rotation of the block groups. In the proposed control strategy, the BCI system recognized the player's mental states (four kinds of motor imagery) and translated them into control commands. The correspondence between MI and control command was determined in the procedure of secondary development of 3D Tetris (Table 1).

In addition, two kinds of blink detected from EOG recordings yielded rotation commands for block group control. The block group could be rotated about the *x*-axis, *y*-axis, and *z*-axis in block group coordinate. We used a double blink to alternate the rotation axis in an *X*-*Y*-*Z* loop, and used a single blink to rotate the block group about a given axis. We adopted the theory of behavior-based control to construct the interactive logic. The part of movement and speed control was described as a finite-state automaton (FSA). We interpreted the FSA as a 5-tuple:  

(4) $$\begin{array}{c} M=\left(\;Q,\Sigma ,\delta ,q,F\;\right), \end{array}$$

where *Q* was a set of states, *q* was a set of initial (or starting) states, *F* was a set of final states, Σ was the input alphabet (a finite, nonempty set of symbols), and *δ* was a partial mapping *δ*(*q*_*t*_, *P*(*t*, *T*_*i*_)) → *q*_*t*+1_ denoting transitions (Table 2).

The block group descended at a constant speed in the 3D game space. Players used mentally generated control to move and rotate the block groups in two dimensions. During the BCI game, *V*_*c*_ meant the current speed of block group, which was the vector sum of *x*-axis and *y*-axis velocities, Δ*V*^*X*^ was the unit increment of speed about *x*-axis, *V*_*c*_ + Δ*V*^*X*^ meant that the speed of the block group increased in direction of the *x*-axis, *V*_*c*_ − Δ*V*^*X*^ meant the speed of the block group decreased in direction of the *x*-axis, and Δ*V*^*Y*^ had the same function in speed adjustment with respect to the *y*-axis. Start was the initial state of all control. Once a new block group appeared at the top of 3D space, the FSA turned to the state N_B (New Block group). So the set of states was {*V*_*c*_ + Δ*V*^*X*^, *V*_*c*_ − Δ*V*^*X*^, *V*_*c*_ + Δ*V*^*Y*^, *V*_*c*_ − Δ*V*^*Y*^, *V*_*c*_ = 0, Start, N_B, Reset}.

We defined the alphabet Σ as {*P*_=_, *P*_+_, *P*_−_ && *V*_*c*_ > 0, Cross, Fallen, Touch, Null, ton, foot, left, right}. Definitions of these symbols are as follows: *P*_=_ meant that the number of a given MI category detected from the EEG within one second (unit time) did not change; *P*_+_ meant that the number increased; *P*_−_  &&  *V*_*c*_ > 0 meant that the number decreased and the current speed was more than zero. There were 20 vertical layers in 3D space. Event outcomes were coded as follows: if the block groups overflowed from 3D space, the Cross outcome turned the FSA to Reset. The code, ton, meant that the FSA received the recognition result, “MI of tongue motion,” as a signal for a state transition. The code, foot, corresponded to “MI of foot motion.” Respectively, left corresponded to “MI of left hand motion” and right corresponded to “MI of right hand motion.” There were four outcome codes: the Touch code meant the Block group touched one of the four standing planes of the 3D game space, while Fallen meant the Block group touched the bottom plane of the 3D space. Cross denoted that the block groups filled the 3D space; then the FSA turned to Reset. NULL meant that the FSA did not receive any directional control commands.

## 3. Results

### 3.1. Characteristic Components

Through the preprocessing of motor imagery training data, we picked up the most suitable characteristic components for the classification of motor imagery described in Table 3. Take Player 1, for example, the characteristic components came from electrode Cz in the 8–12 Hz frequency band, electrode C3 in the 12–16 Hz frequency band, electrode Fz in the 14–16 Hz frequency band, electrode F4 in the 20–22 Hz frequency band, and electrode T7 in the 24−26 Hz frequency band. After gaining all players characteristic components, we carried out filtering operation as Table 3 for preprocessed EEG data. The selected characteristic components would be used in offline algorithm training.

### 3.2. Multifeature Extraction

**Figure 5:**
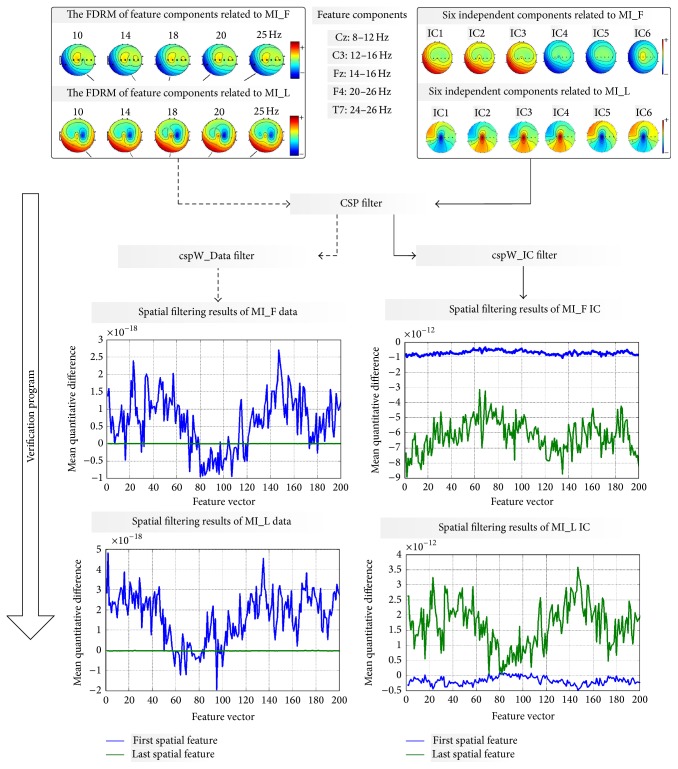
Comparisons of results from cspW_Data and cspW_IC. The upper left part is the frequency domain relief topographic map (FDRM) of feature components relevant to the motor imagery of foot (MI_F) and left hand (MI_L). The upper right part is the frequency domain relief map of independent components relevant to the motor imagery of foot and left hand.

We took Player 1 as example to interpret the output of the verification program (Figure 5), and illustrate how the proposed ICA (retaining the temporal structure of EEG signals) impacted common spatial features positively.

The CSP spatial filters trained from two kinds of components were called cspW_Data and cspW_IC, respectively. The lower left part of Figure 5 illustrates the quantitative difference between the first and last feature components extracted from cspW_Data. The mean quantitative difference relevant to the motor imagery of foot was 0.78 × 10^−18^, and it was 1.26 × 10^−18^ relevant to the motor imagery of left hand. The lower right part illustrates the difference between the first and last feature components extracted from cspW_IC. The mean quantitative difference relevant to the motor imagery of foot was 0.51 × 10^−12^, and it was 1.97 × 10^−12^ relevant to the motor imagery of left hand. For Player 1, compared from the angle of order of magnitude, cspW_IC produced more prominent quantitative differences between spatial features extracted from two kinds of motor imagery signals.

### 3.3. Pattern Discrimination

To verify the effectiveness of EEG features extracted by multifeature extraction, we compared the performances on EEG data for each player among SWNN, RBF neural network, BP neural network, and least squares support vector machines (LS-SVM) techniques. The average accuracy or error rate was over 10 runs of the 10 × 10-fold cross-validation procedure. We implemented the LS-SVM multiclass with one versus one decomposition strategy, using MATLAB (ver. 7.7, R2009b) using the LS-SVMlab toolbox (Version 1.8). The details about parameter setting for these three algorithms and algorithm toolboxes using are in the literature (Table 4) [44].

### 3.4. Control Task

In the control task, ten players were divided into two equal sized groups. One group (Group S) experienced the traditional asynchronous BCI paradigm. The other group (Group 3D) experienced the 3D Tetris paradigm. Group S contained Player 1 (S1), Player 2 (S2), Player 3 (S3), Player 4 (S4), and Player 5 (S5). Group 3D contained Player 6 (3D_1), Player 7 (3D_2), Player 8 (3D_3), Player 9 (3D_4), and Player 10 (3D_5). All players went through the given paradigm for 10 runs in one day. The control task lasted ten days.

For Game-BCI 3D Tetris, the rules and mechanisms were described in Sections 2.3.2 and 2.5. A single run in this pattern started from player's Start command by pressing the button “Game Start.” Once the state of Cross occurred, the single run ended. If, during a given run, the player made one layer of Block-heap disappear, the player scored one point. The player's final score for a given test day was the average score over 10 runs. We used the daily scores as the evaluation criterion of the player's spontaneous ERD production ability.

**Figure 6:**
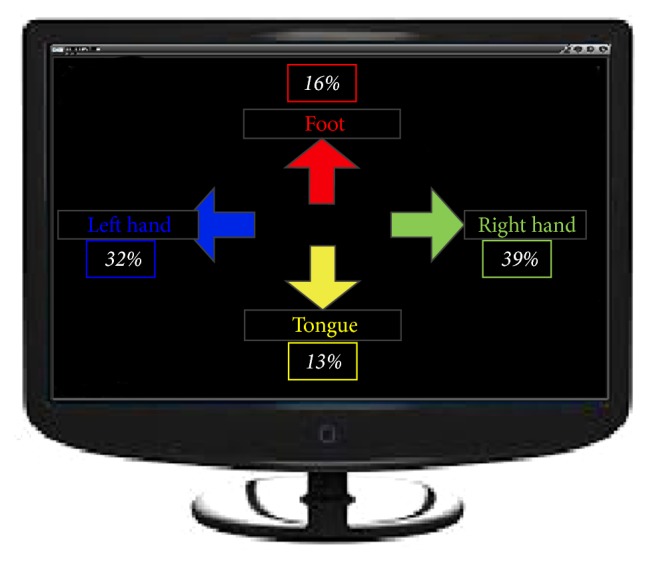
Screen Game Scene.

The traditional asynchronous BCI paradigm used as contrast experiment in this paper was called the Screen Game; it ran in a 2D environment (Figure 6). We collected EEG recordings as described in Section 2.2. The calculation flow of EEG signal processing started from preprocessing steps mentioned in Section 2.4. With multifeature extraction, CSP spatial filtering used the independent components as inputs. The classifier was SWNN. Here, no control strategy functioned in the game. The feedback of one kind of motor imagery was shown on the screen as a percentage number, which was the ratio of its frequency of occurrence to the total number of times during certain time period (the average amount of time taken to complete 3D Tetris single run). The objective of this game was for players to produce ERD features to balance four percentage numbers relevant to different motor imagery categories. The standard deviation of these four percentage numbers was the evaluation criterion. Decreasing standard deviations across days indicated improvement.

#### 3.4.1. Significance Analysis of ERD/ERS

**Figure 7:**
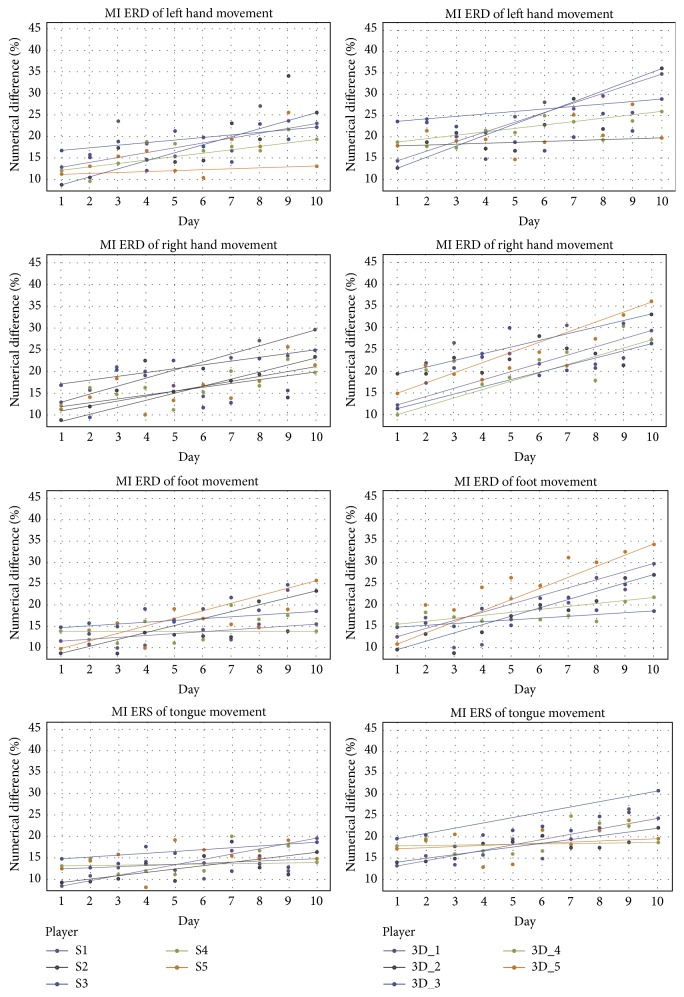
ERD/ERS produced by players in the two games used in the experiment across 10 test days.

Just as prior knowledge of the physiological processes underlying motor imagery does, hand motor imagery will stimulate the electroactivities focusing on contralateral regions over the motor cortex area containing Mu or Beta event-related desynchronization (ERD) and ipsilateral event-related synchronization (ERS) activity. Both ERD and ERS patterns localizing in the midcentral or parietal area are significant for the foot motor imagery. Otherwise, only ERS activity in this area is sufficiently dominant for tongue motor imagery [44]. With two different experimental paradigms and EEG calculating processes, we extracted ERD/ERS features related to MI. Using the EEG power spectrum in the idle state as the benchmark, we compared the mean quantitative differences between idle state and MI (Figure 7).

In Figure 7, each line represents a single player: left column, Screen Game (2D) environment; right column, 3D Tetris environment. Each point is the mean performance on a given day and each line represents the overall trend of the mean numerical differences over 10 training days.

We performed a 2 (groups: Group S, Group 3D) × 10 (test days) two-way ANOVA, with repeated measures over day, on these quantitative differences. The main effect for days was significant, *F* = 2.427, *P* < 0.005, but the main effect for groups was not significant, *F* = 0.850, *P* = 0.207. There was a statistically significant group × day interaction, *F* = 3.643, *P* = 0.014. A simple main effects test for days occurred for Group S subjects, *F* = 4.213, *P* = 0.0584; Tukey's HSD test for multiple comparisons revealed significant improvement in ERD values between day 1 and day 4 (*P* = 0.0427) and increasing tendency among day 4 and day 10 (*P* = 0.074). A simple main effect for days was also found for Group 3D players, *F* = 7.302, *P* = 0.012. Tukey's HSD test for multiple comparisons revealed significant improvement in ERD values between day 1 and day 4 (*P* = 0.026) and increasing tendency among day 4 and day 10 (*P* = 0.003).

In order to investigate the impact of individual variability on the effect of ERD/ERS, we applied Welch's *t*-test on the ERD/ERS quantitative differences of individual players in Groups S and 3D between day 1 and day 10. We found that three players in Group 3D showed statistically significant improvements, *P* = 0.02, *P* < 0.05, and *P* < 0.001. No subjects showed statistical significance in Group S. After 10 training days, the group that performed MI in the 3D Tetris environment showed significant improvement in generating MI-associated ERD/ERS compared with the group in the Screen Game environment. That result suggested that an immersive and rich-control environment for MI would improve the associated mental imagery and enhance MI-based BCI skills.

#### 3.4.2. Game Score

In this research, though 3D Tetris brought the entirely different operating experiences to players compared to 2D Screen Game and a lot of incomparable elements existed between these two BCI paradigms, they all were the method to test the player's spontaneous ERD/ERS production ability.

**Figure 8:**
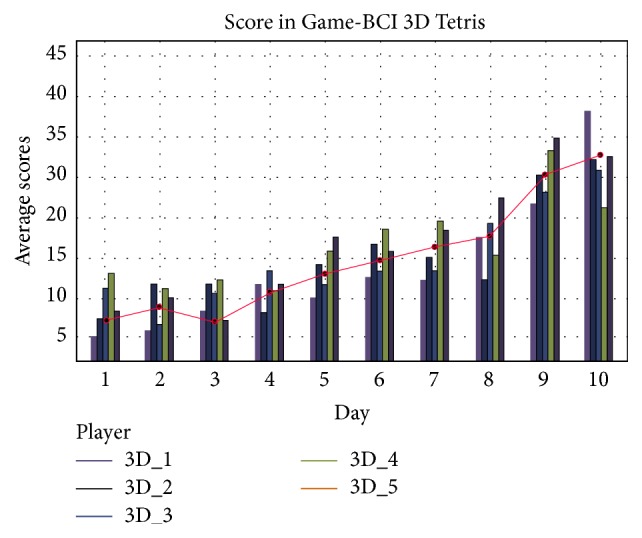
Distribution of players' scores from training day 1 to day 10 in 3D Tetris Game-BCI.

In the 3D Tetris Game-BCI, the score represented the number of layers of disappearing Block-heaps. So a higher score represented a better ability to control the block objects using mind control. From training day 1 to day 4, players' scores did not show an upward trend, *P* = 0.066. However, from training day 5 to day 10, an obvious uptrend in scores appeared, *P* < 0.005 (Figure 8).

So we separated the 10 training days into two stages: Stage I (S_I) covered from day 1 to day 4 and Stage II  (S_II)  covered from day 5 to day 10. The details of the 3D Tetris Game-BCI experiment were described in Table 5. The first four rows represented the mean numbers of motor imagery commands used in two stages. The row labeled “Single blink EOG” and “Double blink EOG” meant the mean number of single blink and double blink commands used in two stages. “Number of Block” was the mean number of block groups. “Mean Duration of a run” meant how long players can remain playing. The experimental data showed that when players obtained higher scores (Stage II), they remained playing for longer. In addition, during 10 training days, the Game-BCI output one MI command in 1.43 seconds (var: ±0.028) averagely.

**Figure 9:**
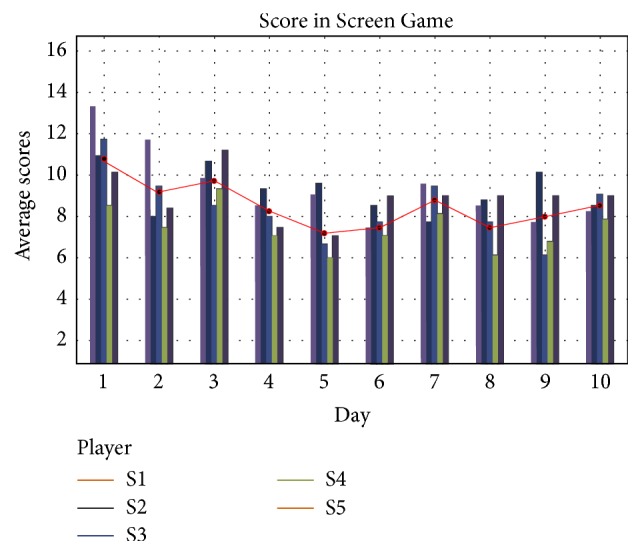
Distribution of players' scores from training day 1 to day 10 in Screen Game.

For the 2D Screen Game, the player's mission was to balance numbers relevant to different motor imagery categories. The score was the standard deviation of these four percentage numbers, which meant that a lower score represented better ability to generate motor imagery. However, from training day 1 to day 10, players' scores did not show an obvious downward trend, *P* = 0.078 (Figure 9).

## 4. Discussion and Conclusion

In this study, we have shown that the combination of video game and BCI is a new design approach to enhance the stimulation and feedback ability of BCI systems. We implemented a Game-BCI system for 3D Tetris game playing with motor imagery indicated by EEG and blink EOG elements. We proposed and tested two key techniques, multifeature extraction and shared control, for enhancing player's BCI control ability, to demonstrate the feasibility that 3D game environment could enhance player's spontaneous ERD/ERS production ability. Taking the 2D Screen Game as a contrast, we compared the quantitative differences between spatial features extracted from motor imagery EEG collected in two experiments separately. The results of the analysis of ERD/ERS and game scores both suggested that an immersive and rich-control environment would improve user's MI ability and BCI control skills.

### 4.1. Multifeature Extraction

The method of multifeature extraction, combining independent component analysis and common spatial patterns, is a renovated mode for EEG feature extraction. Independent component analysis (ICA) is a standard tool for data analysis in the area of neural networks and signal processing. The typical application is blind source separation of EEG signals. In raw EEG signals, there are electrooculograms, electromyography, and other artifacts, as well as mutual interferences between signals. The most direct phenomenon is the submergence of small power components exported from other leads, when there is a large power component from a given lead. Extraction via decorrelation of independent components in a multilead time domain with mixed signals could help indicate the energy distribution of each independent component during a certain period or a special cerebral state. The identification of temporal independence is one part of EEG signal processing. Spatial features illustrate EEG expressions of various mental tasks from the perspective of time-varying features of signal energy in the whole brain. In this way, unlike the extraction of time domain features, the spatial domain emphasizes spatial correlations among original signals or among certain types of components. Instead of merely analyzing energy features of a single channel EEG signal, the algorithm considering frequency spectrum variation correlations between different channels facilitates the creation of connections between EEG feature distribution and complex mental tasks. The common spatial pattern method (CSP), based on the theory of matrix simultaneous diagonalization, involves searching for a set of spatial filters, under the effects of which the variance of one type of signal reaches a maximum and the other type of signal reaches a minimum, thereby achieving classification. Because the EEG variance within a specific frequency band is related to its signal energy, the common spatial pattern method was able to achieve optimal classification of EEG signals based on waveband energy features.

In this study, we applied a time model-based residual mutual information minimization independent source signal extraction method based on artifact elimination and characteristic component extraction of EEG signal of limb motor imagery. This method reduces the correlations components under conditions of preserving temporal structures of EEG signals and so provides clear observation of signal characteristics of each component.

To validate the efficiency of multifeature extraction, two computation processes were derived. The spatial filter cspW_Data was trained with feature components. After multifeature extraction, the spatial filter trained with independent components was called cspW_IC. The results of spatial filtering demonstrated that, compared to cspW_Data, cspW_IC could produce more prominent quantitative differences between spatial features extracted from different motor imagery signals.

### 4.2. 3D Tetris BCI Game

In this research, as a means to assess the utility of the MI control methodology we developed, we integrated BCI design into a 3D Tetris game. The goal was to improve the function in motor imagery training of the BCI system. This attempt follows the idea of gamification for rehabilitation highly respected frontiers. Studies under this new concept, which wants to gamify the process of rehabilitation, have gained wider attention in the rehabilitation field. For example, the Wellapets video game helps teach children how to manage asthma [45]. The social game, Keas, is the leading Health Management platform for employers [46]. The Kognito Co. developed an educational role-playing game to help parents to discuss the underage drinking problem with their children [47]. Run an Empire, a very representative augmented reality game, lets users through “running” way to create their own territory [48]. The goal of systems mentioned above is to help make rehabilitation environments more engaging and more applicable.

Rehabilitation is complex. It involves an ever-changing interaction of the rehabilitation patient with different clinical environments and healthcare providers. It has gone beyond simply creating a fun and exciting application in which to complete rehabilitation exercises and interventions. A delicate balance of the task and the patient's abilities must be achieved. For BCI systems, the created system should be usable across experimental paradigms and at different phases in the rehabilitation training process. Sollfrank et al. [49] showed that a realistic visualization in 3D of upper and lower limb movements can amplify motor related potentials better than in 2D visualization during subsequent motor imagery. Cho and Lee [50] implemented a real-time game environment system using game player's emotional state information from the BCI sensor to raise the degree of immersion in an FPS game. Kondo and colleagues [51] investigated the effect of static and dynamic visual representations of target movements during BCI neurofeedback training, which revealed that dynamic images showed significant improvement in generating MI-associated ERD compared with static images. Belkacem et al. [52] presented real-time control of a video game with eye movements for an asynchronous and noninvasive communication system using two temporal EEG sensors. EEG-controlled gaming applications allow BCI to provide not only entertainment but also strong motivation for practicing, thereby achieving better control with rehabilitation systems.

In our research, the game part contained more of a gambling element compared to the Game-BCI system above. The 3D visual environment did not completely immerse players but felt more like an operating space. Players paid most attention in the ERD/ERS pattern generation. In order to make players feel that they were completing a complicated control mission with four motor imagery and two EOG commands, an interpretation method of physiological signal was formed based on the concept of shared control. Through evaluating the significance of ERD/ERS generation, we found that 3D Tetris Game-BCI provided an effective approach for players to enhance MI-based BCI skills. During 10 training days, the rapid growth of scoring rate appeared in the last five days. We interpret that outcome to mean that players were willing to use the 3D Tetris Game-BCI system after they mastered the needed skills. So we claim that the pattern of Game-BCI will be a tremendous advance in BCI research field.

## Figures and Tables

**Table 1:** The correspondence between motor imagery, object control command, and game effect.

Motor imagery	Control command	3D Tetris coordinate
Foot motion	Moving to Foot Plane	Positive *y*-axis
Tongue motion	Moving to Tongue Plane	Negative *y*-axis
Left hand motion	Moving to Left Plane	Positive *x*-axis
Right hand motion	Moving to Right Plane	Negative *x*-axis

**Table 2:** State transition for movement and speed control.

Input	Current state					
	Start/*V*_*c*_ = 0	*V*_*c*_ + Δ*V*^*x*^	*V*_*c*_ − Δ*V*^*x*^	*V*_*c*_ + Δ*V*^*Y*^	*V*_*c*_ − Δ*V*^*Y*^	N_B
Left	*V*_*c*_ + Δ*V*^*x*^		*V*_*c*_ + Δ*V*^*x*^	*V*_*c*_ + Δ*V*^*x*^	*V*_*c*_ + Δ*V*^*x*^	
Right	*V*_*c*_ − Δ*V*^*x*^	*V*_*c*_ − Δ*V*^*x*^		*V*_*c*_ − Δ*V*^*x*^	*V*_*c*_ − Δ*V*^*x*^	
Tongue	*V*_*c*_ − Δ*V*^*Y*^	*V*_*c*_ − Δ*V*^*Y*^	*V*_*c*_ − Δ*V*^*Y*^	*V*_*c*_ − Δ*V*^*Y*^		
Foot	*V*_*c*_ + Δ*V*^*Y*^	*V*_*c*_ + Δ*V*^*Y*^	*V*_*c*_ + Δ*V*^*Y*^		*V*_*c*_ + Δ*V*^*Y*^	
*P*_=_		*V*_*c*_ + Δ*V*^*x*^	*V*_*c*_ − Δ*V*^*x*^	*V*_*c*_ + Δ*V*^*Y*^	*V*_*c*_ − Δ*V*^*Y*^	
*P*_+_		*V*_*c*_ + Δ*V*^*x*^	*V*_*c*_ − Δ*V*^*x*^	*V*_*c*_ + Δ*V*^*Y*^	*V*_*c*_ − Δ*V*^*Y*^	
*P*_−_ && *V*_*c*_ > 0		*V*_*c*_ + Δ*V*^*x*^	*V*_*c*_ − Δ*V*^*x*^	*V*_*c*_ + Δ*V*^*Y*^	*V*_*c*_ − Δ*V*^*Y*^	
Touch	*V*_*c*_ = 0	*V*_*c*_ = 0	*V*_*c*_ = 0	*V*_*c*_ = 0	*V*_*c*_ = 0	*V*_*c*_ = 0
Fallen		N_B	N_B	N_B	N_B	
Cross	Reset	Reset	Reset	Reset	Reset	Reset
*Null*						*V*_*c*_ = 0

**Table 3:** The frequencies and electrodes of all feature components.

Player	Electrode	Frequency	[*R*^2^: mean ± var]
Player 1	Cz	8–12 Hz	[0.49 ± 0.024]
	C3	12–16 Hz	[0.48 ± 0.032]
	Fz	14–16 Hz	[0.35 ± 0.03]
	F4	20–22 Hz	[0.26 ± 0.022]
	T7	24–26 Hz	[0.23 ± 0.032]

Player 2	C4	16–20 Hz	[0.49 ± 0.03]
	Cz	20–24 Hz	[0.38 ± 0.025]
	C3	24–26 Hz	[0.32 ± 0.031]
	F4	10–12 Hz	[0.30 ± 0.042]
	T3	24–28 Hz	[0.22 ± 0.02]

Player 3	C4	16–18 Hz	[0.43 ± 0.024]
	Cz	20–24 Hz	[0.42 ± 0.04]
	C3	26–28 Hz	[0.40 ± 0.048]
	P3	18–22 Hz	[0.37 ± 0.01]
	Pz	10–18 Hz	[0.32 ± 0.024]

Player 4	C4	20–16 Hz	[0.49 ± 0.024]
	F3	12–10 Hz	[0.37 ± 0.01]
	C3	20–22 Hz	[0.32 ± 0.01]
	T3	22–26 Hz	[0.32 ± 0.022]
	Cz	14–16 Hz	[0.26 ± 0.024]

Player 5	Cz	10–14 Hz	[0.58 ± 0.062]
	F3	18–22 Hz	[0.37 ± 0.050]
	C4	20–24 Hz	[0.37 ± 0.075]
	T7	8–14 Hz	[0.34 ± 0.700]
	C3	10–14 Hz	[0.21 ± 0.062]

Player 6	C4	12–16 Hz	[0.47 ± 0.022]
	Cz	20–24 Hz	[0.36 ± 0.032]
	Fz	24–26 Hz	[0.36 ± 0.059]
	C3	8–16 Hz	[0.35 ± 0.03]
	F7	22–24 Hz	[0.3 ± 0.042]

Player 7	Cz	10–12 Hz	[0.52 ± 0.062]
	Pz	20–26 Hz	[0.44 ± 0.070]
	C4	22–24 Hz	[0.33 ± 0.055]
	C3	10–14 Hz	[0.31 ± 0.700]
	T8	10–12 Hz	[0.28 ± 0.062]

Player 8	C4	16–22 Hz	[0.49 ± 0.03]
	Cz	20–24 Hz	[0.48 ± 0.042]
	Pz	20–24 Hz	[0.44 ± 0.032]
	Fz	16–22 Hz	[0.44 ± 0.031]
	F4	10–18 Hz	[0.37 ± 0.05]

Player 9	C4	18–24 Hz	[0.55 ± 0.03]
	Cz	22–28 Hz	[0.52 ± 0.01]
	C3	24–28 Hz	[0.38 ± 0.032]
	Pz	18–22 Hz	[0.42 ± 0.03]
	P3	22–26 Hz	[0.33 ± 0.01]

Player 10	Fz	10–18 Hz	[0.43 ± 0.024]
	C3	18–22 Hz	[0.42 ± 0.04]
	T4	24–28 Hz	[0.41 ± 0.048]
	C4	26–28 Hz	[0.32 ± 0.01]
	F3	10–14 Hz	[0.32 ± 0.024]

**Table 4:** The mean accuracy of classification from four classifiers based *on two kinds* of feature extraction.

	SWNN (mean)		RBF (mean)		BP (mean)		LS-SVM (mean)	
	cspW_Data	cspW_IC	cspW_Data	cspW_IC	cspW_Data	cspW_IC	cspW_Data	cspW_IC
Player 1	87.10	86.6	78.61	85.2	82.74	80.6	68.37	72.0
Player 2	79.66	82.9	72.11	74.72	75.90	77.5	71.64	68.0
Player 3	65.29	74.0	83.67	76.1	62.37	72.8	67.20	72.2
Player 4	76.40	76.4	66.81	67.51	59.31	71.2	71.59	70.4
Player 5	60.80	63.6	59.72	53.92	61.54	63.3	58.20	59.4
Player 6	74.60	78.5	66.27	77.2	54.87	74.6	62.81	67.5
Player 7	56.30	76.3	49.52	74.97	72.10	69.6	52.61	60.1
Player 8	66.94	81.3	49.83	79.30	53.30	72.8	57.22	62.0
Player 9	72.13	77.45	65.81	73.62	65.26	77.3	63.70	68.95
Player 10	71.16	83.6	50.6	82.0	57.0	75.1	59.77	74.7
Mean	71.03	***78.7***	64.3	***74.5***	64.4	***73.5***	63.3	***67.5***
*P* value	**0.008**		**0.042**		**0.038**		**0.019**	

The classification results from four classifiers indicated that cspW_IC produced more quality features than cspW_Data. To investigate the statistical significance of the accuracies, we performed an analysis of variance (ANOVA) on each player's result based on all classification accuracies (10 runs of the 10 × 10-fold cross-validation procedure). The *P*-value from SWNN was 0.008, 0.042 from RBF neural network, 0.038 from BP neural network, and 0.019 from LS-SVM. These *P*-values were leass than 0.05 for all players, which indicated that the difference was significant.

**Table 5:** The details of the 3D Tetris Game-BCI experiment.

	3D_1		3D_2		3D_3		3D_4		3D_5	
	S_I	S_ II	S_I	S_II	S_I	S_II	S_I	S_II	S_I	S_II
Number of right hand MI	52	76	32	83	89	173	87	183	72	176
Number of left hand MI	41	33	25	95	82	116	95	106	68	188
Number of Foots MI	38	44	47	66	71	105	114	127	92	109
Number of Tongue MI	21	35	22	56	79	119	73	98	64	124
Single blink EOG	33	40	30	46	36	70	52	62	42	77
Double blink EOG	47	49	26	34	18	26	18	19	12	21
Number of Block	31	96	48	102	51	132	74	101	42	94
Mean Duration of a run	477 s	1440 s	720 s	1530 s	754 s	1980 s	1260 s	1710 s	630 s	1880 s

## Appendix

The algorithm called one-versus-rest (OVR) CSP is an extension of a well-known method called common spatial patterns to multiclass case, to extract signal components specific to one condition from electroencephalography (EEG) data sets of multiple conditions.

In this research, the details of the one-versus-rest CSP algorithm are as follows.

*E*_*R*_, *E*_*L*_, *E*_*F*_, and *E*_*T*_ represented the matrix of independent components (temporal features) related to right hand motor imagery, left hand motor imagery, foot motor imagery, and tongue motor imagery with dimensions *N* × *T*. *N* was the number of independent components, and *T* is the number of sampling points. The normalized spatial covariance of the independent source signals could be represented as *C*_*R*_, *C*_*L*_, *C*_*F*_, and *C*_*T*_. The composite spatial covariance could be factorized as

(A.1) $$\begin{array}{c} C_{C}=C_{R}+C_{L}+C_{F}+C_{T}=U_{C}\lambda_{C}U_{C}^{T}. \end{array}$$

Here, *U*_*C*_ was the matrix of eigenvectors and *λ*_*C*_ was the diagonal matrix of eigenvalues. $P=\sqrt{\lambda_{C}^{-1}}U_{C}^{T}$ denoted the whitening transformation matrix. To see how to extract common spatial patterns specific to right hand motor imagery, let *C*_*R*_′ = *C*_*L*_ + *C*_*F*_ + *C*_*T*_. Then *C*_*R*_′ and *C*_*R*_ are individually transformed as

(A.2) SR=PRRPT,SR′=PRR′PT.

Here, *S*_*R*_ and *S*_*R*_′ share the same eigenvector and the sum of corresponding eigenvalues for two matrices is always one. It can be understood that if *S*_*R*_ can be factored as

(A.3) $$\begin{array}{c} S_{R}=U\Lambda_{R}U^{T} \end{array}$$

then

(A.4) SR′=UΛR′UT,

(A.5) ΛR+ΛR′=I.

Combine (A.2), (A.3), and (A.4) and then obtain

(A.6) ΛR=PTUTRRPTU=SFRRRSFRTΛR′=PTUTRR′PTU=SFRRR′SFRT,

where *SF*_*R*_ = *U*^*T*^*P*. Note that, from (A.5), it is obvious that larger eigenvectors corresponding to larger eigenvalues yield a high variance under condition “right hand motor imagery” and a low variance under other conditions (other kinds of motor imagery). With the projection matrix *W*_*R*_ = *U*^*T*^*P* we can get *Z*_*R*_ = *W*_*R*_*E*_*R*_. Repeating the procedure, we can obtain *Z*_*L*_, *Z*_*F*_, and *Z*_*T*_.

However, the variances of only a small number *m* of the spatial filtered signals were used as input features for classification. The *m* first rows of *Z*_*R*_ formed the feature vector *Z*_*I*_ given by the equation

(A.7) $$\begin{array}{c} Z_{I}=\log\left(\;\frac{var\left(Z_{I}^{\prime }\right)}{\sum_{i=1}^{m}var\left(Z_{I}^{\prime }\right)}\;\right). \end{array}$$
